# Sodium dichloroacetate exhibits anti-leukemic activity in B-chronic lymphocytic leukemia (B-CLL) and synergizes with the p53 activator Nutlin-3

**DOI:** 10.18632/oncotarget.2018

**Published:** 2014-05-26

**Authors:** Chiara Agnoletto, Elisabetta Melloni, Fabio Casciano, Gian Matteo Rigolin, Erika Rimondi, Claudio Celeghini, Laura Brunelli, Antonio Cuneo, Paola Secchiero, Giorgio Zauli

**Affiliations:** ^1^ Department of Morphology, Surgery and Experimental Medicine and LTTA Centre, University of Ferrara, Ferrara, Italy; ^2^ Department of Medical Sciences, University of Ferrara-Arcispedale S.Anna, Ferrara, Italy; ^3^ Department of Life Sciences, University of Trieste, Trieste, Italy; ^4^ Institute for Maternal and Child Health, IRCCS “Burlo Garofolo”, Trieste, Italy

**Keywords:** Sodium dichloroacetate, Nutlin-3, B-CLL, p21

## Abstract

The anti-leukemic activity of the mitochondria-targeting small molecule sodium dichloroacetate (DCA), used alone and in association with the small molecule inhibitor of the p53/MDM2 interaction Nutlin-3, was analyzed in primary B-chronic lymphocytic leukemia (B-CLL) samples (n=22), normal peripheral blood cells (n=10) and in p53^wild-type^ EHEB, JVM-2, JVM-3 B lymphoblastoid cell lines. DCA exhibited a dose-dependent anti-leukemic activity in both primary B-CLL and B leukemic cell lines with a functional p53 status and showed a synergistic cytotoxic activity when used in combination with Nutlin-3. At the molecular level, DCA positively regulated p53 activity, as documented by post-transcriptional modifications of p53 protein, and synergized with Nutlin-3 in increasing the expression of the p53-target genes *MDM2*, *PUMA*, *TIGAR* and in particular *p21*. The potential role of p21 in mediating the DCA+Nutlin-3 anti-leukemic activity was underscored in knocking-down experiments. Indeed, transfection of leukemic cells with p21 siRNAs significantly decreased the DCA+Nutlin-3-induced cytotoxicity. Taken together, our data emphasize that DCA is a molecule that merits to be further evaluated as a chemotherapeutic agent for B-CLL, likely in combination with other therapeutic compounds.

## INTRODUCTION

Most cancers possess a near-universal metabolic phenotype known as the ‘Warburg effect’, which is characterized by enhanced glycolytic flux for ATP production, glucose to lactate conversion and reduced mitochondrial oxidative phosphorylation, even under aerobic conditions [1-3]. There is now growing evidence that the mitochondria might be primary targets in cancer therapeutics instead of simple bystanders during cancer development. Interestingly, this cancer-specific metabolic remodeling can be reversed by dichloroacetate (DCA), a mitochondria-targeting small molecule able to penetrate most tissues after oral administration [4]. DCA shows several interesting characteristics, such as effectiveness in a variety of solid tumor cell lines and relatively low toxicity on normal cells. Moreover, DCA is a generic drug with low price, which has been in use in humans for more than 30 years. Most of the current studies on DCA have been performed on solid tumors, such as non-small cell lung cancer, breast cancer and most glioblastoma cell lines [4,5]. Additional studies have been performed on endometrial, prostate, breast and colorectal [6-14] cancer cells and more recently on multiple myeloma cells [15]. On the other hand, since leukemic cell lines use glycolysis but also non-glucose bioenergetic pathways such as fatty acid oxidation and/or amino-acid metabolism [16], the potential efficacy of DCA on leukemic cells should be investigated, also considering that no data are currently available on this issue.

On these bases, the aim of the present study was to evaluate the potential therapeutic perspectives of DCA as anti-leukemic drug. For this purpose, DCA was added to primary B chronic lymphocytic leukemia (B-CLL) cells and to primary normal peripheral blood mononuclear cells (PBMC), either alone or in association with Nutlin-3, a small molecule inhibitor of the MDM2/p53 interaction. In addition, the molecular mechanisms of action of DCA+Nutlin-3 were evaluated in a panel of p53^wild-type^ B leukemic cell lines (EHEB, JVM-2, JVM-3).

## RESULTS

### DCA promotes cytotoxicity in primary B-CLL patient derived cells, but not in normal peripheral blood cells

**Figure 1:**
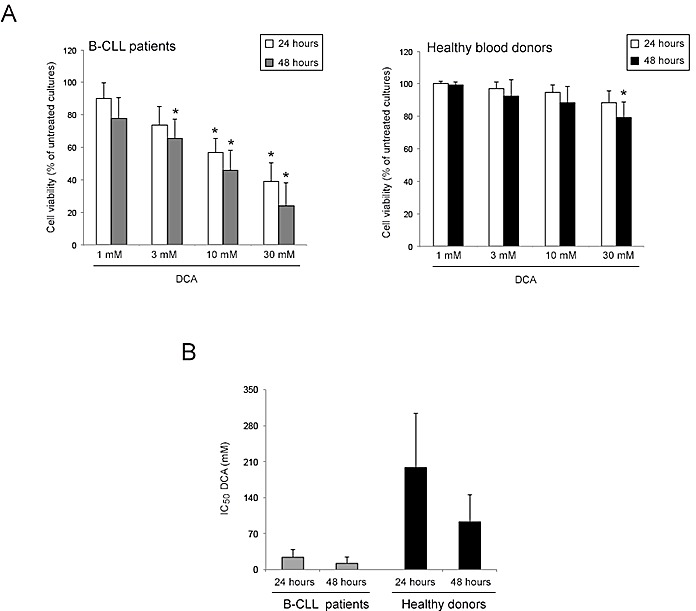
Comparative evaluation of cell viability in response to DCA in B-CLL patient leukemic cells and PBMC from healthy human blood donors B-CLL patient cell samples (n=22) and PBMC from healthy blood donors (n=10) were exposed to serial doses of DCA (range 1-30 mM) as indicated. In A, cell viability was analyzed at 24 and 48 hours of treatment and was calculated as percentage of untreated cultures set to 100%. Data are reported as mean values ±SD. The asterisk indicates p<0.05 with respect to the untreated cultures. In B, IC_50_ values are reported as mean value ±SD.

In the first group of experiments, the effect of DCA was comparatively analyzed on primary PBMC derived from B-CLL patients (n=22; Table 1) and from healthy blood donors (n=10). Treatment with DCA, used in the range of 1-30 mM for up to 48 hours, exhibited a dose- and time-dependent cytotoxicity, resulting in significant reduction of leukemic cell viability with respect to the untreated cultures, at concentrations ≥3 mM in B-CLL patient cell samples (Figure 1A). The IC_50_ mean values (±SD) of cytotoxicity of DCA in B-CLL patient samples were 24±15 mM and 12±12 mM at 24 and 48 hours of treatment, respectively (Figure 1B). On the other hand, PBMC obtained from healthy donors were significantly less susceptible to DCA cytotoxicity as compared to primary B-CLL cells, with IC_50_ mean values (±SD) of cytotoxicity of 198±106 mM and 93±52 mM at 24 and 48 hours of treatment (Figure 1A-B), clearly showing that normal PBMC were completely resistant to DCA effects at concentrations ≤10 mM. Thus, in line with previous data obtained in solid tumor cell models [4-14] and multiple myeloma [15], we have demonstrated for the first time that DCA promoted a significant cytotoxicity also in primary B-CLL samples but not in normal PBMC.

**Table 1:** Clinical and laboratory features of the B-CLL patients involved in this study

Patient #	Age/Sex	Rai stage	WBC count (x103/mL)	%CD38+ cells	ZAP-70	IgVH status	Cytogenetic abnormalities (FISH)	p53 status	Therapy
1	77/M	0	81.4	2	2	mut	del13q omoz.	WT	none
2	85/F	1	54	1	5	mut	tri12	WT	Chl
3	68/M	0	82.9	3.3	21	mut	del13q	na	none
4	79/F	1	47.9	2.9	na	na	na	na	FCR
5	75/F	1	56.6	1.9	8	mut	na	na	none
6	73/F	1	95	2.1	50	mut	del13q	WT	Chl
7	75/M	0	96.8	1.1	30	mut	del13q	WT	none
8	79/F	0	54.8	6.3	1.8	unmut	del11q/del13q omoz.	WT	F
9	66/M	0	108.1	0	24.3	unmut	del13q	WT	FCR
10	56/M	II	128.3	1.2	60	unmut	Normal	WT	FCR
11	82/F	0	23.3	50.9	6.4	mut	Normal	WT	none
12	55/F	0	9.3	77.9	13	unmut	del11q	WT	none
13	65/M	0	25.3	6	27.1	mut	Normal	WT	none
14	75/M	0	23.1	21	19.7	unmut	del13q	WT	none
15	73/M	0	27.7	10.3	10.2	mut	del13q	WT	none
16	71/F	0	14.2	0	20.6	na	del13q	WT	none
17	75/M	0	59.7	6.8	0.3	mut	del13q	WT	none
18	55/F	0	86.5	7	7.4	unmut	del13q	WT	none
19	77/M	IV	100	15.4	72.1	unmut	tris 12	WT	Chl
20	51/F	0	105	2.8	20	unmut	del11q/del13q	WT	none
21	87/F	II	121	58.5	2.4	unmut	del13q/tris 12	WT	Chl
22	72/M	II	21.2	55.2	na	unmut	del13q/tris 12	WT	none

FCR, fludarabine, cyclophosphamide, rituximab; Chl, chlorambucil; mut, mutated; unmut, unmutated; WT, wild-type; na, not available.

### DCA activates the p53 pathway in p53^wild-type^ B leukemic cell lines

**Figure 2:**
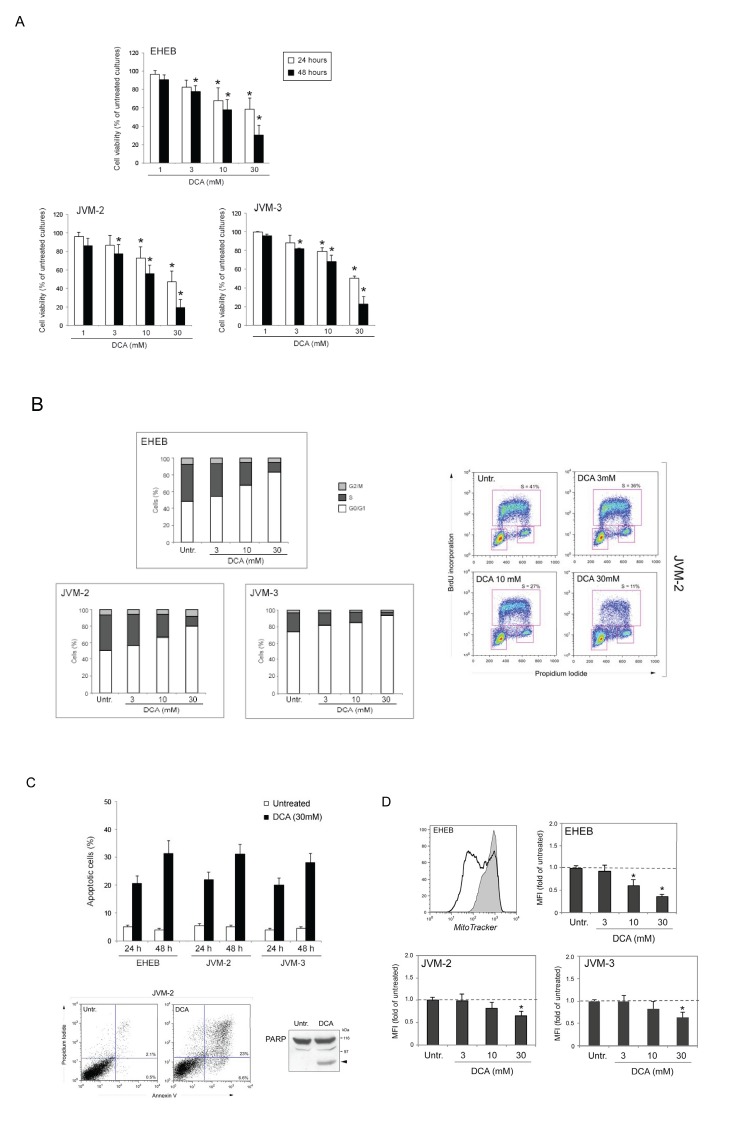
Cytotoxicity induced by DCA in B leukemic cell lines The p53^wild-type^ (EHEB, JVM-2, JVM-3) B leukemic cell lines were exposed to DCA before analysis of cell toxicity. In A, cell viability in response to serial doses of DCA (range 1-30 mM), was calculated at both 24 and 48 hours of treatment as percentage with respect to the control vehicle cultures (set to 100% for each cell line). In B, cell distribution in the different phases of cell cycle was calculated from the flow cytometry dot plots after BrdU/PI staining and expressed as percentage of the total population. Representative cell-cycle profiles of cells, either left untreated or treated with serial doses of DCA, analyzed by flow cytometry are shown. In the right panel, the rectangles represent the cells in G0/G1, S, G2/M phases of the cell cycle. In C, the percentage of apoptotic cells was determined by flow-cytometry after Annexin V/PI staining (upper panel). A representative flow cytometric analysis of apoptosis, validated also by Western blot analysis of PARP cleavage, is shown (lower panels). In D, to measure changes in the mitochondrial membrane potential (ΔY_m_), upon treatment with DCA for 24h cells were stained with MitoTracker solution and analyzed by flow cytometry. Median values of mitochondrial MitoTracker fluorescence and representative histograms are shown. In A, C and D, data are reported as the mean±SD of results from three independent experiments. In A and D, the asterisk indicates p<0.05 with respect to the untreated cultures of each cell line.

Since most B-CLL at diagnosis are p53^wild-type^ [17,18], a condition also characterizing the primary B-CLL samples analyzed in this study, it is noteworthy that previous studies on solid tumors have implicated the p53 pathway in mediating the anti-tumoral activity of DCA [5,6,19]. Therefore, in order to investigate the potential involvement of p53 in mediating the anti-leukemic activity of DCA, we next performed a series of experiments on three p53^wild-type^ B leukemic cell lines (EHEB, JVM-2 and JVM-3), used as model systems. As shown in Figure 2A, treatment with DCA resulted in a dose-dependent reduction of cell viability in all the p53^wild-type^ B leukemic cell lines tested, with IC_50_ values ranging from 25 to 30.4 mM at 24 hours of treatment and from 15.2 to 18.6 mM at 48 hours of treatment, comparable to those of primary B-CLL cells (Figure 1B). Flow cytometric analysis of these leukemic cell cultures demonstrated that treatment with DCA induced early accumulation in G1 phase of the cell cycle (Figure 2B) and promoted apoptosis in all leukemic cell lines (Figure 2C). The cytotoxic effects of DCA on the B cell lines were coupled to the ability of DCA to reduce the glucose consumption (data not shown) and the mitochondrial membrane potential (Figure 2D).

**Figure 3:**
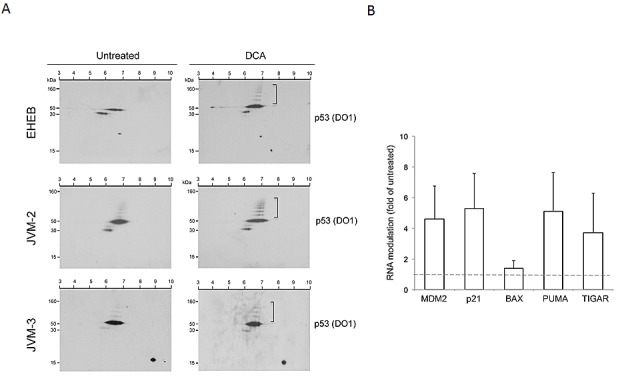
Activation of p53 pathway by DCA in p53^wild-type^ B leukemic cells p53^wild-type^ B leukemic EHEB, JVM-2 and JVM-3 cells were left untreated or treated with DCA for 24 hours. In A, cell lysates were analyzed by two-dimensional immunoblot. The antibody p53 (DO-1) identified a spot group corresponding to full-length p53 (~53 kDa) and a series of p53 isoforms with a molecular weight above 53 kDa (square brackets). In B, transcriptional activation of p53 target genes, *MDM2*, *p21*, *BAX*, *PUMA* and *TIGAR*, was assessed by quantitative RT-PCR. mRNA levels are expressed as folds of modulation, with respect to the control untreated cultures set at 1. Results are reported as means±SD of six independent experiments carried out on the three different cell lines.

Based on these evidences, documenting the anti-proliferative/pro-apoptotic activity of DCA in B leukemic cells, we next assessed the effect of DCA treatment on the expression of p53 in the EHEB, JVM-2, JVM-3 cell lines by two-dimensional electrophoresis and immunoblotting, which allows separation of proteins based on their molecular weight (MW) and isoelectric point (pI). As shown in Figure 3A, two-dimensional electrophoresis revealed the presence of the full-length p53 (_~_53 kDa, pI 6-7.3) beside a group of spots below the 53 kDa (_~_40 kDa, pI 5.5-6.5) in the untreated cultures. Upon DCA treatment for 24 hours, it was possible to observe a slight increase in the full-length p53 protein in the EHEB cell line and, of note, the appearance of a series of isoforms above the 53 kDa in all the cell lines, suggestive of p53 post-translational modifications. In this respect, in Western blot assay we have documented that DCA dose-dependently induced p53 phosphorylation in Ser^15^ and Ser^392^ (data not shown). To test the hypothesis of functional activation of p53 upon exposure to DCA, in parallel experiments we have analyzed the levels of a subset of p53 transcriptional targets involved in promoting cell cycle arrest (p21), modulation of apoptosis (MDM2, Bax, PUMA) or metabolism (TIGAR) by RT-PCR. As shown in Figure 3B, DCA variably but significantly (>2 fold) increased the mRNA levels of *MDM2*, *p21*, *PUMA* and *TIGAR* in all leukemic cell lines investigated, while no significant modulation of *BAX* mRNA was observed.

### DCA plus Nutlin-3 combination exhibits a potent synergistic anti-leukemic activity

**Figure 4:**
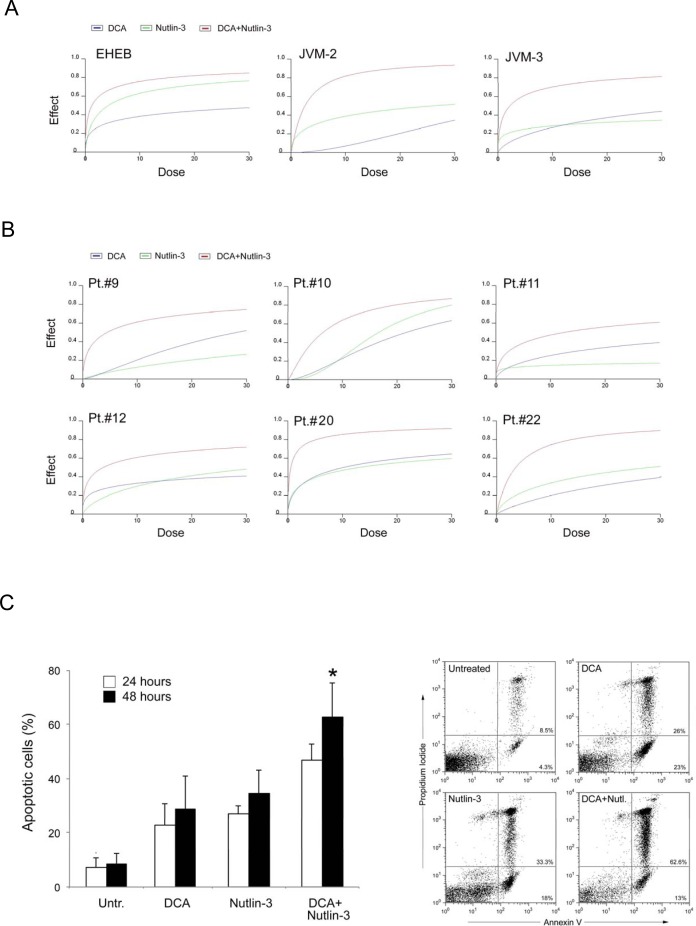
Cytotoxicity by DCA and Nutlin-3 used alone or in combination in p53^wild-type^ B leukemic cells and B-CLL patient leukemic cells p53^wild-type^ B leukemic EHEB, JVM-2 and JVM-3 cells and B-CLL patient leukemic cells were exposed to serial doses of DCA or Nutlin-3 used either alone or in combination, with a fixed ratio, for 24 hours. Dose-effect plots, to determine drug efficacy, are shown for each cell line (A) and for representative B-CLL patient samples (B). The decrease of cell viability, labeled “effect” on the Y-axis, was determined in assays done at least twice in duplicate. In C, induction of apoptosis in B-CLL patient leukemic cells was calculated as percentage of Annexin V/PI cells. Data are reported as mean±SD of results from three independent experiments. A representative flow cytometric analysis of apoptosis in a primary B-CLL sample is shown. The asterisk indicates p<0.05 with respect to cultures treated with either DCA or Nutlin-3 alone.

In the next group of experiments, we have examined the potential interaction of DCA with Nutlin-3, a small molecule which transcriptionally activates p53 by abolishing its interaction with its principal inhibitor MDM2 [20]. For this purpose, leukemic cells were treated with DCA (3-30 mM) and Nutlin-3 (1-10 μM), used as single agents and in combination. In particular, B-CLL leukemic cells were treated with serial concentrations of DCA and Nutlin-3 at a constant DCA:Nutlin-3 ratio for data analysis by the method of Chou and Talalay [21]. Combined treatment with DCA+Nutlin-3 resulted in significantly (p<0.01) greater cytotoxicity with respect to the single agents, with a synergistic effect both in primary B-CLL patient samples as well as in B leukemic cell lines (Figure 4A-B), as documented by an average Combination Index (CI) <1 (Table 2). The cytotoxicity induced by the combined treatment with DCA+Nutlin-3 was mainly due to the increase of the degree of apoptosis with respect to the treatment with DCA or Nutlin-3 used alone (Figure 4C).

**Table 2:** Combination index values for the effect of DCA plus Nutlin-3 on viability of B-CLL patient cells and p53wild-type B cell lines

Cells	ED50	ED75	ED90	Average CI
Patient #1	0.33	0.29	0.26	0.29
Patient #2	0.18	0.03	0.01	0.07
Patient #3	0.37	0.38	0.4	0.38
Patient #4	0.31	0.39	0.52	0.41
Patient #5	0.43	0.37	0.34	0.38
Patient #6	0.37	0.31	0.30	0.33
Patient #7	0.37	0.32	0.29	0.33
Patient #8	0.06	0.16	0.46	0.23
Patient #9	0.2	0.56	1.91	0.79
Patient #10	0.67	0.95	1.36	0.99
Patient #11	0.20	0.27	0.37	0.28
Patient #12	0.15	0.29	0.68	0.37
Patient #13	0.05	0.06	0.09	0.07
Patient #14	0.19	0.02	0.004	0.07
Patient #15	0.35	0.1	0.03	0.16
Patient #16	0.24	0.04	0.007	0.10
Patient #17	0.11	0.30	0.80	0.40
Patient #18	0.45	0.43	0.42	0.43
Patient #19	0.01	0.08	0.58	0.22
Patient #20	0.02	0.10	0.67	0.26
Patient #21	0.22	0.24	0.29	0.25
Patient #22	0.19	0.13	0.09	0.14
EHEB	0.39	0.49	0.66	0.51
JVM-2	0.16	0.11	0.13	0.13
JVM-3	0.06	0.08	0.11	0.08

ED indicates effect dose. The average combination index (CI) values were calculated from ED50, ED75, and ED90.

### Role of p21 induction in DCA and DCA+Nutlin-3 induced cytotoxicity in leukemic cells

**Figure 5:**
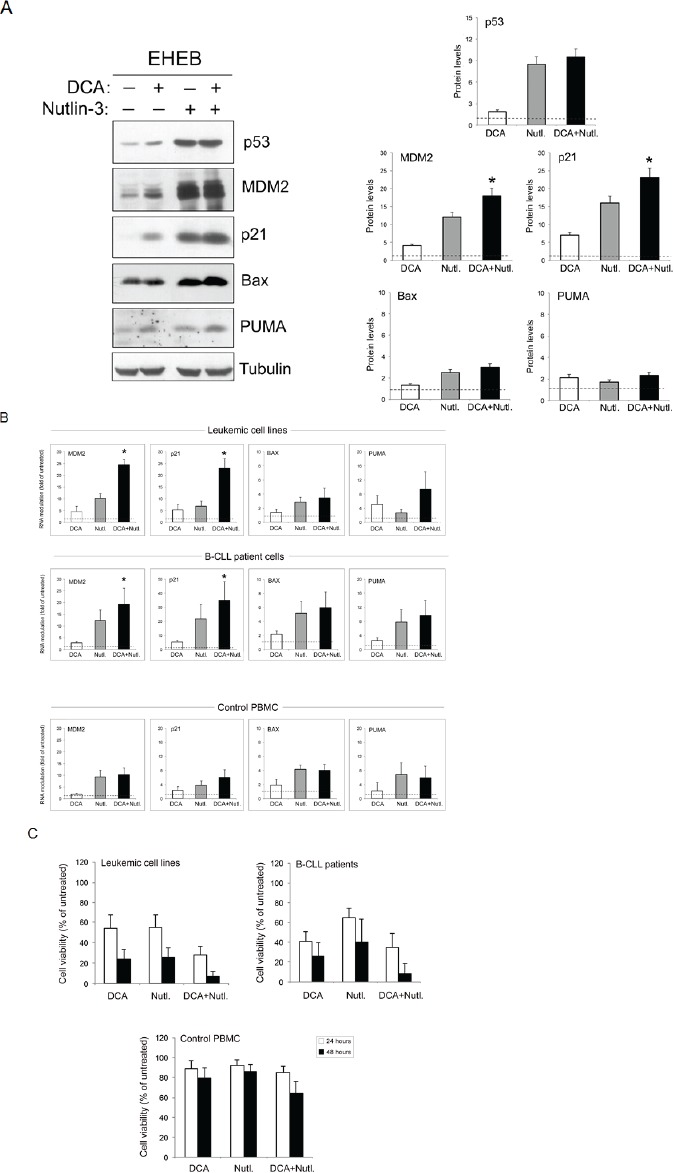
Activation of p53 pathway by DCA+Nutlin-3 combination In A, equal amounts of cell lysates, obtained from EHEB cells treated for 24 hours with DCA and Nutlin-3, used either alone or in combination, were analyzed for protein levels by Western blot. Tubulin staining is shown as a loading control. Blots representative of at least three independent experiments yielding equivalent results are shown. After densitometric analyses, protein levels are expressed as folds of protein modulation, by the indicated treatments, with respect to the control untreated cultures set at 1. In B and C, p53^wild-type^ B leukemic cell lines, B-CLL patient leukemic cells and PBMC from healthy human blood donors respectively were exposed for 24 hours to DCA and Nutlin-3, used either alone or in combination, as indicated. In B, the expression levels of p53 target genes were assessed by quantitative RT-PCR and results were indicated as folds of modulation with respect to the control untreated cultures set at 1. Data are reported as mean±SD of results from independent experiments. The asterisk indicates p<0.05 with respect to cultures treated with either DCA or Nutlin-3 alone. In C, cell viability was analyzed at 24 and 48 hours of treatment and was calculated as percentage of untreated cultures set to 100%. Data are reported as mean values ±SD.

With respect to DCA treatment, Nutlin-3 induced a much more potent accumulation of both p53 and its molecular targets MDM2 and p21 in p53^wild-type^ leukemic cells, as documented by Western blot analysis performed on total cell lysates (Figure 5A). The combination of DCA+Nutlin-3 did not result in a further accumulation of p53 protein with respect to Nutlin-3 alone while it promoted a greater induction of MDM2 and p21 proteins with respect to Nutlin-3 alone (Figure 5A). Interestingly, the combination of Nutlin-3+DCA significantly (p<0.05) enhanced the induction of *MDM2* and *p21* at the mRNA level, in both B leukemic cell lines and primary B-CLL patient samples, but not in normal PBMC (Figure 5B). Of note, the synergistic transcriptional induction of p53-target genes by DCA+Nutlin-3, documented in leukemic cells but not in normal PBMC, correlated with the lower cytotoxicity of DCA+Nutlin-3 in these cells (Figure 5C).

**Figure 6:**
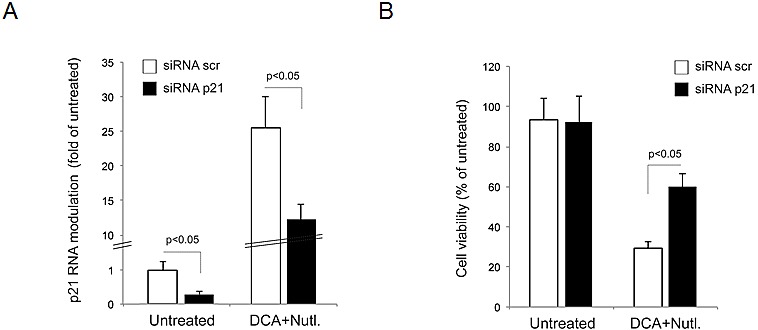
Role of p21 pathway in mediating the anti-leukemic activity of DCA+Nutlin-3 EHEB cells were transfected with either control scrambled (scr) siRNA or p21 siRNA before treatment. In A, after transfection, efficiency of p21 knock-down was documented by analyzing levels of *p21* mRNA by quantitative RT-PCR, in untreated cells and upon treatment with DCA+Nutlin-3. Results are expressed as folds of modulation with respect to the control cultures. In B, cultures transfected with either control scrambled (scr) siRNA or p21 siRNA were analyzed for cell viability upon exposure to DCA+Nutlin-3. Results are expressed as percentage with respect to the control vehicle cultures (set to 100%). Data are reported as means±SD.

Starting from the data illustrated above, which demonstrate that DCA+Nutlin-3 strongly synergized both in promoting leukemic cytotoxicity as well as in inducing p53 transcriptional activity, it was particularly noteworthy that one of the genes up-regulated in response to DCA±Nutlin-3 was *p21*. In this respect, a recent study has demonstrated that a strong induction of p21 by genotoxic agents was predictive of better prognosis in B-CLL [22]. Conversely, a lack of induction of p21 was observed in B-CLL patient samples showing a worse prognosis even in the presence of an integral activation of p53 [22]. Therefore, in order to functionally elucidate the role of p21 in mediating the anti-leukemic activity of DCA+Nutlin-3, we utilized siRNAs to attenuate p21 expression. The knock-down of *p21* expression was demonstrated by quantitative RT-PCR, documenting a significant reduction of *p21* mRNA in EHEB cells, transfected with a cocktail of p21 specific siRNAs, both at baseline as well as after DCA+Nutlin-3 treatment (Figure 6A). Of note, in EHEB samples in which p21 expression was knocked-down by transfection with p21 specific siRNAs, the DCA+Nutlin-3-mediated cytotoxicity was significantly (p<0.05) reduced with respect to either cells transfected with a scrambled control siRNA or cells not transfected (Figure 6B). Overall, these experiments support a potential role of p21 in modulating the anti-leukemic activity of DCA+Nutlin-3.

## DISCUSSION

In this study, we have demonstrated for the first time that DCA exerts cytotoxic activity in primary B-CLL patient samples, but not in normal PBMC obtained from healthy individuals, in the same range of concentrations previously used in studies performed on solid tumors and on multiple myeloma [4-15] and that are therapeutically achievable *in vivo*. Although the cytotoxic activity of DCA has been attributed to PDK inhibition in solid tumor cell models [1], the concentrations of DCA required to induce apoptosis and/or to inhibit cell proliferation *in vitro* in B leukemic cells are several log greater than the inhibition constant (Ki) of PDK. Similar data were previously obtained in studies performed in solid tumors [4-15]. In considering the importance of our current demonstration that DCA shows an anti-leukemic activity, it should be noticed that there are 40 years of human experience with mechanistic studies of DCA in human tissues after oral use, pharmacokinetic and toxicity data from randomized studies [23]. This supports an easy translation to early phase clinical trials. In fact, DCA is an orally bioavailable, low-cost small molecule and currently being evaluated in phase I/II clinical trials for glioblastoma, gliomas and other solid tumors [24]. DCA has interesting characteristics as anticancer drug since it is easily administered and affordable.

By using as a model system a panel of p53^wild-type^ B leukemic cell lines, we demonstrated that DCA induces both cell cycle arrest and apoptosis. Thus, although it has been previously hypothesized that the lack of mitochondrial hyperpolarization in certain types of cancers, including hematological malignancies [25], might render DCA ineffective in such cases, and although the relation between mitochondrial depolarization and cytotoxicity varies in different tumor cell models [26], we found that DCA induced mitochondrial depolarization in B leukemic cell lines. In any case, it should be taken into account that a metabolic heterogeneity has been documented in another model of hematological malignancy, such as multiple myeloma [15] as well as in leukemic cell lines [16].

At the molecular level, DCA induced post-transcriptional modifications of p53 with activation of the p53 transcriptional activity, as documented by the induction of canonical p53 target genes, such as *MDM2, p21* and *PUMA* but not of *BAX*. Another major finding of our study was the ability of DCA to potently synergize with Nutlin-3, a non-genotoxic activator of the p53 pathway, in promoting cytotoxicity in B leukemic cell lines as well as in primary B-CLL patient cells, with significantly less toxicity on normal PBMC. The contribution of the transcriptional activity of p53 in promoting leukemic cytotoxicity is still under debate, as transcriptional independent activities have also been demonstrated in previous studies performed with Nutlin-3 [17,18]. However, our current data demonstrate the existence of a correlation between the ability to promote the transcription of p53 transcriptional targets, and in particular *p21*, and the synergistic cytotoxic activity in response to the DCA+Nutlin-3 treatment. Indeed, while the DCA+Nutlin-3 combination was extremely efficient in inducing the transcription of *p21* in both primary B-CLL patient samples and B leukemic cell lines, it was significantly less efficient when added to primary normal PBMC. Interestingly, our current demonstration that DCA+Nutlin-3 potently synergized in inducing the transcriptional activation of *p21* is noteworthy since the transcriptional induction of *p21* has been involved in mediating the therapeutic effect of chemotherapeutic drugs in B-CLL [22]. In this respect, the key role of p21 in mediating the anti-leukemic activity of DCA+Nutlin-3 was confirmed in p21 knock-down experiments. In considering the cytotoxic activity of DCA+Nutlin-3 in p53^wild-type^ B-CLL cells, it is noteworthy that rapamycin, like DCA, decreases the Warburg effect by inhibiting the mTOR pathway [27] and that also Nutlin-3 via p53 can inhibit the mTOR pathway [28,29]. Thus, besides inducing p21, it is possible that inhibition of the mTOR pathway also contributes to the cytotoxicity of the DCA+Nutlin-3 combination.

Since p53 defects are detected in less than 10% of B-CLL patients at diagnosis [17,18], our study suggests that the anti-leukemic therapeutic potential of DCA, either used alone or in combination with Nutlin-3, is promising and should be further investigated since the majority of B-CLL are suitable to respond to the DCA+Nutlin-3 combination. With respect to the subset of p53^mutated^ B-CLL cells, which significantly increases in B-CLL refractory to fludarabine treatment [17,18], it should be explored the possibility that the DCA+Nutlin-3 combination might benefit from the addition of a microtubule active drug, like vinblastine or nocodazole [30-34]. This hypothesis will require further investigations in future studies.

## METHODS

### Primary B-CLL patient samples and B leukemic cell lines

For experiments with primary cells, peripheral blood samples were collected in heparin-coated tubes from 22 B-CLL patients and 10 healthy blood donors following informed consent, in accordance with the Declaration of Helsinki and in agreement with institutional guidelines (University-Hospital of Ferrara). The main clinical parameters of the B-CLL patients were abstracted from clinical records (Table 1). All patients had been without prior therapy at least for three weeks before blood collection.

Peripheral blood mononuclear cells (PBMC) were isolated by gradient centrifugation with lymphocyte cell separation medium (Cedarlane Laboratories, Hornby, ON). T lymphocytes, NK lymphocytes, granulocytes and monocytes were negatively depleted from peripheral blood B-CLL with immunomagnetic microbeads (MACS microbeads, Miltenyi Biotech, Auburn, CA), with a purity >95% of resulting CD19^+^ population, as assessed by flow cytometry analysis and previously described [35]. Primary cells were cultured in RPMI-1640 medium containing 10% FBS, L-glutamine and penicillin/streptomycin (all from Gibco, Grand Island, NY).

The p53^wild-type^ B leukemic cell lines EHEB, JVM-2, JVM-3 [36] were purchased from DSMZ (Deutsche Sammlung von Mikroorganismen und Zellkulturen GmbH, Braunschweig, Germany) and were routinely cultured in RPMI-1640 supplemented with 10% FBS, L-glutamine and penicillin/streptomycin (all from Gibco).

### Culture treatments, assessment of cell viability, apoptosis, cell cycle profile and glucose consumption

For *in vitro* treatments with DCA (Sigma-Aldrich, St Louis, MO), used either alone or in combination with Nutlin-3 (Cayman Chemicals, Ann Arbor, MI), cells were seeded at a density of 1×10^6^ cells/ml and cultured under normoxic conditions. At different time points after treatment, cell viability was examined by Trypan blue dye exclusion and MTT (3-(4,5-dimethilthiazol-2yl)-2,5-diphenyl tetrazolium bromide) colorimetric assay (Roche Diagnostics Corporation, Indianapolis, IN) for data confirmation, as previously described [37]. Levels of apoptosis were quantified by Annexin V-FITC/propidium iodide (PI) staining (Immunotech, Marseille, France) followed by analysis using a FACSCalibur flow cytometer (Becton-Dickinson, San Jose, CA). To avoid non-specific fluorescence from dead cells, live cells were gated tightly using forward and side scatter, as described [38, 39]. The cell cycle profile was analyzed by flow cytometry after 5-bromodeoxyuridine (BrdU) incorporation. Briefly, leukemic cells were incubated with 50 μM BrdU (Sigma-Aldrich) at 37°C for 1 hour. The antibody anti-BrdU (BD Biosciences Pharmingen, San Diego, CA) was bound to BrdU incorporated into neosynthesized DNA and the complex was detected by FITC conjugated secondary antibody (Immunotech). Cells were then stained with PI (50 μg/mL) and analyzed by flow cytometry as previously described [40]. In parallel, glucose consumption was measured as the conversion of glucose to 6-phosphogluconate and NADH with the Glucose (HK) Assay Kit (Sigma-Aldrich), as indicated by the manufacturer and previously described [41].

### Flow cytometric assessment of mitochondrial activity

Mitochondrial activity was evaluated staining cells with MitoTracker® Green^FM^ (Molecular Probes, Inc., Eugene, Oregon), which passively diffuses across the plasma membrane and accumulates in active mitochondria. Briefly, at different time points, cells were incubated with pre-warmed MitoTracker staining solution (obtained diluting MitoTracker® Green^FM^ in serum-free medium to a final concentration of 25 nM) for 30 minutes at 37°C. Cells were then washed with PBS (Gibco) and analyzed by flow cytometry as previously described [42].

### Western blot analyses

For Western blot analysis, cells were lysed as previously described [43-44]. Protein determination was performed by using the BCA Protein Assay (Thermo Scientific, Rockford, IL). Samples were supplemented with loading buffer (250 mM Tris pH 6.8, 2% SDS, 10% glycerin, 4% beta-mercaptoethanol, 1% bromophenol blue) and boiled for 2 minutes. Equal amounts of protein for each sample were migrated in SDS-polyacrylamide gels and blotted onto nitrocellulose filters, as previously described [43,44]. The following Abs were used: anti-p53 (DO-1), anti-MDM2 (SMP14), anti-p21 (C-19), anti-PUMAα/β (H-136), anti-PARP-1 (H-250) and anti-Bax (2D2) purchased from Santa Cruz Biotechnology (Santa Cruz, CA); anti-Phospho-p53 (Ser15) and anti-Phospho-p53 (Ser392) from Cell Signaling Technology (Danvers, MA); anti-tubulin from Sigma-Aldrich. After incubation with anti-mouse or anti-rabbit IgG horseradish peroxidase-conjugated secondary Abs (Sigma-Aldrich), specific reactions were revealed with the ECL Lightning detection kit (Perkin Elmer, Waltham, MA). Densitometry values for Western blot were estimated by the ImageQuant TL software (GE Healthcare, Buckinghamshire, UK) and were expressed as arbitrary units (a.u.). Multiple film exposures were used to verify the linearity of the samples analyzed and to avoid saturation of the film.

### Bi-dimensional gel electrophoresis (2-DE) and immunoblotting

For bi-dimensional gel electrophoresis, protein extraction was performed by adding 150 μl of lysis buffer (5M urea, 2M tiourea, 2% CHAPS, 2% Zwittergent detergent; all from Calbiochem, San Diego, CA) with protease inhibitors (Complete, mini EDTA-free mixture, Roche Applied Science, Milan, Italy) to the cell pellets. Lysates were sonicated for 3 min, and 250 units of benzonase endonuclease (Novagen, San Diego, CA) were added to each sample and incubated for 40 minutes at room temperature on rotary shaker. Cell debris were removed by centrifugation at 13000 × g for 10 minutes, 15°C. The supernatants were aliquoted and stored at −80°C. Protein concentration was measured with a modified Bradford method (BioRad, Milan, Italy). Protein extracts (30 μg) from each sample were diluted to a final volume of 125 μl in the rehydration solution (5M urea, 2M thiourea, 2% CHAPS, 2% Zwittergent, 100 mM DeStreak, 0.5% IPG buffer pH 3–10 linear; all from GE Healthcare) and then applied on immobilized pH 3–10 linear gradient strips, 7 cm (IPG strips, GE Healthcare). Briefly, IPG strips were hydrated on an IPGphor apparatus (GE Healthcare) for 16 h at 30 V/h and then focused for 26 h until 50,000 V-h. After the first-dimension run, proteins were reduced by LDS Sample Buffer (Life Technologies, Carlsbad, CA) containing 60 mM DTT (GE Healthcare). Proteins were alkylated by 100 mM iodoacetamide (Sigma-Aldrich). The strips were then embedded in 0.7% (w/v) agarose on the top of 1-mm-thick acrylamide precast gels at 10% (Life Technologies). After electrophoresis the proteins were transferred to nitrocellulose membrane by standard electroblotting and stained with MemCode™ Reversible protein stain kit (Fisher Scientific, Illkirch Cedex, France). After blocking with 5% dry milk in TBS-Tween20 for 1 h at room temperature, the membranes were probed with an anti-p53 (DO-1) antibody (Santa Cruz Biotechnology) and then exposed to the peroxidase-linked specie specific anti-mouse IgG (Sigma-Aldrich). The p53 positive proteins were visualized using chemiluminescence plus (ECL plus) Western blotting detection reagents (GE Healtcare).

### RNA analyses

Total RNA was extracted from cells using the QIAGEN RNeasy Plus mini kit (QIAGEN, Hilden, Germany) according to the supplier's instructions. Once verified the quality of RNA preparation by agarose gel, total RNA was transcribed into cDNA, using the QuantiTect^®^ Reverse Transcription kit (QIAGEN). *p21*, *MDM2*, *BAX*, *PUMA*, *TIGAR* gene expression was analyzed using the SYBR Green-based real-time quantitative polymerase chain reaction (RT qPCR) detection method with SABiosciences RT^2^ Real- Time™ Gene expression assays, which include specific validated primer sets and PCR master mix (SABiosciences, QIAGEN). All samples were run in triplicate using the real time thermal analyzer Rotor-Gene™ 6000 (Corbett, Cambridge, UK), as previously described [45,46]. Expression values were normalized to the housekeeping gene *POLR2A* amplified in the same sample.

### Transfection experiments

Leukemic cells (2×10^6^ cells/0.1 ml) were mixed with either 1 μg of control enhanced green fluorescence protein (EGFP) plasmid or 2 μg of small interfering RNA (siRNA) cocktails, transferred to a 2.0-mm electroporation cuvette and nucleofected with the nucleofector kit V (Lonza Cologne AG, Cologne, Germany) using the nucleofector device (Amaxa Nucleofector II apparatus, Lonza). After electroporation, cells were immediately transferred to a complete medium and cultured at 37°C until analysis. Transfection efficiency was monitored in each experiment by scoring the percentage of EGFP-positive cells by flow cytometry analysis. For specific p21 gene knock-down, siRNAs were designed and manufactured by Ambion^®^ (Life Technologies) according to the current guidelines for effective gene knock-down by this method. Negative control siRNA consisted of a 19 bp-scrambled sequence with 3' dT overhangs (Ambion's Silencer negative control siRNA). The Ambion's Silencer negative control siRNA sequence has no significant homology to any known gene sequences from human and it has been previously tested for the lack of non-specific effects on gene expression.

### Statistical analysis and assessment of the effect of combination treatment

The results were evaluated by using analysis of variance with subsequent comparisons by Student's t-test and with the Mann-Whitney rank-sum test. Statistical significance was defined as p<0.05. In order to investigate the effect of DCA+Nutlin-3 combination, leukemic cells were then treated with serial doses of DCA (range 3-30 mM) or Nutlin-3 (range 1-10 microM), individually or in combination using a constant ratio (DCA:Nutlin-3). Results were analyzed with the method of Chou and Talalay to determine whether combined treatment yields greater effects than expected from summation alone: a combination index (CI) of 1 indicates an additive effect, while a CI below 1 indicates synergism. For this purpose cell viability data were analyzed with the CalcuSyn software (Biosoft, Cambridge, UK) and reported either as CI values or as dose-effect curves directly drawn by the CalcuSyn software.
